# Neuraminidase inhibitors for preventing and treating influenza in healthy adults and children

**DOI:** 10.1590/1516-3180.20141324T2

**Published:** 2014-05-20

**Authors:** Tom Jefferson, Mark A. Jones, Peter Doshi, Chris B. Del Mar, Rokuro Hama, Matthew Thompson, Elizabeth A. Spencer, Igho Onakpoya, Kamal R. Mahtani, David Nunan, Jeremy Howick, Carl J. Heneghan

## Abstract

**BACKGROUND:**

Neuraminidase inhibitors (NIs) are stockpiled and recommended by public health agencies for treating and preventing seasonal and pandemic influenza. They are used clinically worldwide.

**OBJECTIVE:**

To describe the potential benefits and harms of NIs for influenza in all age groups by reviewing all clinical study reports of published and unpublished randomised, placebo-controlled trials and regulatory comments.

**METHODS:**

*Search methods:* We searched trial registries, electronic databases (to 22 July 2013) and regulatory archives, and corresponded with manufacturers to identify all trials. We also requested clinical study reports. We focused on the primary data sources of manufacturers but we checked that there were no published randomised controlled trials (RCTs) from non-manufacturer sources by running electronic searches in the following databases: the Cochrane Central Register of Controlled Trials (CENTRAL), MEDLINE, MEDLINE (Ovid), EMBASE, Embase.com, PubMed (not MEDLINE), the Database of Reviews of Effects, the NHS Economic Evaluation Database and the Health Economic Evaluations Database.

*Selection criteria:* Randomised, placebo-controlled trials on adults and children with confirmed or suspected exposure to naturally occurring influenza.

*Data collection and analysis:* We extracted clinical study reports and assessed risk of bias using purpose-built instruments. We analysed the effects of zanamivir and oseltamivir on time to first alleviation of symptoms, influenza outcomes, complications, hospitalisations and adverse events in the intention-to-treat (ITT) population. All trials were sponsored by the manufacturers.

**MAIN RESULTS:**

We obtained 107 clinical study reports from the European Medicines Agency (EMA), GlaxoSmithKline and Roche. We accessed comments by the US Food and Drug Administration (FDA), EMA and Japanese regulator. We included 53 trials in Stage 1 (a judgement of appropriate study design) and 46 in Stage 2 (formal analysis), including 20 oseltamivir (9623 participants) and 26 zanamivir trials (14,628 participants). Inadequate reporting put most of the zanamivir studies and half of the oseltamivir studies at a high risk of selection bias. There were inadequate measures in place to protect 11 studies of oseltamivir from performance bias due to non-identical presentation of placebo. Attrition bias was high across the oseltamivir studies and there was also evidence of selective reporting for both the zanamivir and oseltamivir studies. The placebo interventions in both sets of trials may have contained active substances.

*Time to first symptom alleviation*. For the treatment of adults, oseltamivir reduced the time to first alleviation of symptoms by 16.8 hours (95% confidence interval (CI) 8.4 to 25.1 hours, P < 0.0001). This represents a reduction in the time to first alleviation of symptoms from 7 to 6.3 days. There was no effect in asthmatic children, but in otherwise healthy children there was (reduction by a mean difference of 29 hours, 95% CI 12 to 47 hours, P = 0.001). Zanamivir reduced the time to first alleviation of symptoms in adults by 0.60 days (95% CI 0.39 to 0.81 days, P < 0.00001), equating to a reduction in the mean duration of symptoms from 6.6 to 6.0 days. The effect in children was not significant. In subgroup analysis we found no evidence of a difference in treatment effect for zanamivir on time to first alleviation of symptoms in adults in the influenza-infected and non-influenza-infected subgroups (P = 0.53).

*Hospitalisations.* Treatment of adults with oseltamivir had no significant effect on hospitalisations: risk difference (RD) 0.15% (95% CI -0.78 to 0.91). There was also no significant effect in children or in prophylaxis. Zanamivir hospitalisation data were unreported.

*Serious influenza complications or those leading to study withdrawal.* In adult treatment trials, oseltamivir did not significantly reduce those complications classified as serious or those which led to study withdrawal (RD 0.07%, 95% CI -0.78 to 0.44), nor in child treatment trials; neither did zanamivir in the treatment of adults or in prophylaxis. There were insufficient events to compare this outcome for oseltamivir in prophylaxis or zanamivir in the treatment of children.

*Pneumonia.* Oseltamivir significantly reduced self reported, investigator-mediated, unverified pneumonia (RD 1.00%, 95% CI 0.22 to 1.49); number needed to treat to benefit (NNTB) = 100 (95% CI 67 to 451) in the treated population. The effect was not significant in the five trials that used a more detailed diagnostic form for pneumonia. There were no definitions of pneumonia (or other complications) in any trial. No oseltamivir treatment studies reported effects on radiologically confirmed pneumonia. There was no significant effect on unverified pneumonia in children. There was no significant effect of zanamivir on either self reported or radiologically confirmed pneumonia. In prophylaxis, zanamivir significantly reduced the risk of self reported, investigator-mediated, unverified pneumonia in adults (RD 0.32%, 95% CI 0.09 to 0.41); NNTB = 311 (95% CI 244 to 1086), but not oseltamivir.

*Bronchitis, sinusitis and otitis media.* Zanamivir significantly reduced the risk of bronchitis in adult treatment trials (RD 1.80%, 95% CI 0.65 to 2.80); NNTB = 56 (36 to 155), but not oseltamivir. Neither NI significantly reduced the risk of otitis media and sinusitis in both adults and children.

*Harms of treatment.* Oseltamivir in the treatment of adults increased the risk of nausea (RD 3.66%, 95% CI 0.90 to 7.39); number needed to treat to harm (NNTH) = 28 (95% CI 14 to 112) and vomiting (RD 4.56%, 95% CI 2.39 to 7.58); NNTH = 22 (14 to 42). The proportion of participants with four-fold increases in antibody titre was significantly lower in the treated group compared to the control group (RR 0.92, 95% CI 0.86 to 0.97, I2 statistic = 0%) (5% absolute difference between arms). Oseltamivir significantly decreased the risk of diarrhoea (RD 2.33%, 95% CI 0.14 to 3.81); NNTB = 43 (95% CI 27 to 709) and cardiac events (RD 0.68%, 95% CI 0.04 to 1.0); NNTB = 148 (101 to 2509) compared to placebo during the on-treatment period. There was a dose-response effect on psychiatric events in the two oseltamivir “pivotal” treatment trials, WV15670 and WV15671, at 150 mg (standard dose) and 300 mg daily (high dose) (P = 0.038). In the treatment of children, oseltamivir induced vomiting (RD 5.34%, 95% CI 1.75 to 10.29); NNTH = 19 (95% CI 10 to 57). There was a significantly lower proportion of children on oseltamivir with a four-fold increase in antibodies (RR 0.90, 95% CI 0.80 to 1.00, I2 = 0%).

*Prophylaxis.* In prophylaxis trials, oseltamivir and zanamivir reduced the risk of symptomatic influenza in individuals (oseltamivir: RD 3.05% (95% CI 1.83 to 3.88); NNTB = 33 (26 to 55); zanamivir: RD 1.98% (95% CI 0.98 to 2.54); NNTB = 51 (40 to 103)) and in households (oseltamivir: RD 13.6% (95% CI 9.52 to 15.47); NNTB = 7 (6 to 11); zanamivir: RD 14.84% (95% CI 12.18 to 16.55); NNTB = 7 (7 to 9)). There was no significant effect on asymptomatic influenza (oseltamivir: RR 1.14 (95% CI 0.39 to 3.33); zanamivir: RR 0.97 (95% CI 0.76 to 1.24)). Non-influenza, influenza-like illness could not be assessed due to data not being fully reported. In oseltamivir prophylaxis studies, psychiatric adverse events were increased in the combined on- and off-treatment periods (RD 1.06%, 95% CI 0.07 to 2.76); NNTH = 94 (95% CI 36 to 1538) in the study treatment population. Oseltamivir increased the risk of headaches whilst on treatment (RD 3.15%, 95% CI 0.88 to 5.78); NNTH = 32 (95% CI 18 to 115), renal events whilst on treatment (RD 0.67%, 95% CI -2.93 to 0.01); NNTH = 150 (NNTH 35 to NNTB > 1000) and nausea whilst on treatment (RD 4.15%, 95% CI 0.86 to 9.51); NNTH = 25 (95% CI 11 to 116).

**AUTHORS’ CONCLUSIONS:**

Oseltamivir and zanamivir have small, non-specific effects on reducing the time to alleviation of influenza symptoms in adults, but not in asthmatic children. Using either drug as prophylaxis reduces the risk of developing symptomatic influenza. Treatment trials with oseltamivir or zanamivir do not settle the question of whether the complications of influenza (such as pneumonia) are reduced, because of a lack of diagnostic definitions. The use of oseltamivir increases the risk of adverse effects, such as nausea, vomiting, psychiatric effects and renal events in adults and vomiting in children. The lower bioavailability may explain the lower toxicity of zanamivir compared to oseltamivir. The balance between benefits and harms should be considered when making decisions about use of both NIs for either the prophylaxis or treatment of influenza. The influenza virus-specific mechanism of action proposed by the producers does not fit the clinical evidence.

**This is the abstract of a Cochrane Review published in the Cochrane Database of Systematic Reviews (CDSR) 2014, issue 4. Art. No.: CD008965. DOI:** 10.1002/14651858.CD008965.pub4 (http://onlinelibrary.wiley.com/doi/10.1002/14651858.CD008965.pub4/abstract). For full citation and authors’ details, see reference 1.

**The full text is freely available from:** http://onlinelibrary.wiley.com/doi/10.1002/14651858.CD008965.pub4/pdf
